# Fatty Left Ventricular Cardiomyopathy: An Under-Diagnosed Disease

**DOI:** 10.5935/abc.20160199

**Published:** 2017-01

**Authors:** Abdalla Elagha, Anthon Fuisz

**Affiliations:** 1 Cairo University Hospitals, Cairo, Egypt / National Heart, Lung and Blood Institute, Bethesda; 2 Westchester Medical Center, New York, NY, USA

**Keywords:** Cardiomyopathy, Dilated, Tomography, X Ray-Computed/methods, Ventricular Dysfunction, Left, Lipoma

During a preoperative cardiac assessment, a 57-year-old asymptomatic female, without known cardiac risk-factors, was found to have LBBB on ECG. Echocardiography revealed dilated cardiomyopathy. To rule-out coronary artery disease, coronary CT-angiography was performed and showed no significant coronary obstruction.

**Figure 1:**
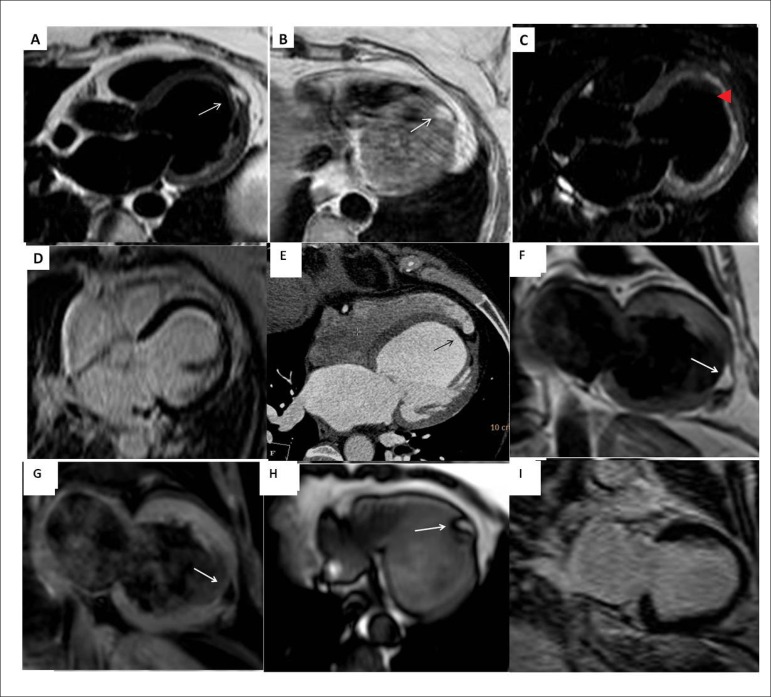
MRI axial images of the heart using different pulse sequences to demonstrate myocardial segment of fatty infiltration in the LV apex. The CMR protocol included T1-weighted spin-echo (with-and-without fat-suppression), T2-weighted black-blood image, and DHE after administration of gadolinium. (A) T2-weighted black blood; (B) T1-weighted turbo spin echo; (C) T1-weighted turbo spin echo with fat saturation (showed clear nulling of apical fatty area); (D) DHE technique showing no evidence of myocardial scar; (E) Multi-detector CT image that shows a focal area of fatty infiltration in the LV apex (black arrow). This area has a negative Hounsfield value indicating its fatty nature. Follow-up 3 Tesla CMR study after 18 months showed no significant changes in fat distribution within LV; (F) Two-chamber T1-weighted turbo spin echo showing the fatty area at the LV apex; (G) Two-chamber T1-weighted turbo spin echo with fat saturation showing clear nulling of apical fatty area; (H) Axial T1-weighted single-shot axial image of the most distal apical portion of LV showing part of the fatty area; (I) DHE technique showing no evidence of myocardial scar; however, the fatty area is not enhanced.

For further evaluation, a cardiac magnetic resonance (CMR) study was performed. A focal area of fatty infiltration was seen in the left ventricular (LV) apex extending from the subendocardial to the subepicardial surfaces (Figure 1A and 1B). This area was suppressed with fat saturation pulse-sequences (Figure 1C), and did not show enhanced signal on delayed-hyperenhancement images (DHE) (Figure 1D). Both LV and LA were dilated; LV systolic function was reduced globally. Additionally, the CMR showed normal RV dimensions, function, and wall-thickness. The fatty area was also seen retrospectively on the cardiac CT (Figure 1E).

A follow-up CMR study after 18 months - using 3 Tesla magnet - showed no significant changes of fat distribution within the LV, and a mild reduction of LV systolic function (ejection fraction =45%) that was similar to the initial study (Figure 1F-I).

There are few differential diagnoses. Firstly, cardiac lipoma, which is usually well-defined and well-encapsulated, and generally produces compression on adjacent cardiac structures. Secondly, fatty deposition following MI is associated with myocardial thinning and scarring on DHE images. Lastly, arrhythmogenic left ventricular cardiomyopathy is a substrate for ventricular arrhythmias and usually involves interventricular septum.

Isolated fatty LV cardiomyopathy is an uncommon clinical entity, and it could be under-diagnosed. With recent technological advancements in CT and MRI, more cases can be detected and investigated.

